# RNA Encapsulation Mode and Evolutionary Insights from the Crystal Structure of Emaravirus Nucleoprotein

**DOI:** 10.1128/spectrum.05018-22

**Published:** 2023-04-11

**Authors:** Lee S. Izhaki-Tavor, Itai G. Yechezkel, Joel Alter, Moshe Dessau

**Affiliations:** a Azrieli Faculty of Medicine in the Galilee, Bar-Ilan University, Safed, Israel; Fujian Agriculture and Forestry University

**Keywords:** *Bunyavirales*, fig mosaic virus, *Fimoviridae*, nucleoprotein, RNA encapsulation, virus evolution, X-ray crystallography, plant viruses, protein-RNA interactions

## Abstract

Enveloped RNA viruses are rare among plant viruses. *Fimoviridae* is a newly founded family of plant viruses within the *Bunyavirales* order that inflicts diverse crop losses worldwide. The fig mosaic virus (FMV), the representative member of the *Fimoviridae* family, was shown to be a causative agent for the fig mosaic disease. Like all bunyaviruses, FMV has a segmented, negative-sense, single-stranded RNA (ssRNA) genome that is encapsulated by the viral nucleoprotein (N). Here, we present high-resolution crystal structures of FMV N in its RNA-free and RNA-bound forms, revealing a “paper fortune teller” structural transition between the two states. The tightly packed tetramer of FNV N is similar to the structures of other N proteins of different members of the *Bunyavirales* order. In its RNA-bound form, the tetramer reorganizes to adopt a more open state that allows the accommodation of the RNA. Despite the low sequence similarity to N proteins of animal-infecting bunyaviruses, there is a striking structural resemblance between FMV N and nucleoproteins from members of the *Peribunyaviridae*, an animal-infecting family of viruses. This structural homology implies that enveloped plant viruses and animal-infecting viruses might have a common ancestor from which they diverged.

**IMPORTANCE** Most insect-born viruses circulate within the *Animalia* kingdom, whereas plant-infecting RNA viruses are cross-kingdom pathogens. Many plant-infecting viruses cause devastating crop damage that leads to food security endangerment. The evolutionary crossroads of interkingdom circulation and infection are poorly understood. Thus, we took the structural approach to understand the similarities and differences between interkingdom-infecting viruses and viruses that circulate within one kingdom of life. Using our structures of FMV N in its free form and in complex with a single-stranded RNA (ssRNA), we dissected the mechanism by which FMV N binds to the RNA and revealed the conformational changes associated with the binding. The resemblance of our structure to N proteins from members of the *Peribunyaviridae* family and their recently published ribonucleoprotein (RNP) pseudoatomic resolution assembly model suggests that the FMV genome is similarly encapsulated. Thus, our finding unveils yet another bridge by which plant- and animal-infecting viruses are interconnected.

## INTRODUCTION

Even though enveloped plant viruses have been recognized for a century, little is known about their assembly’s molecular mechanisms, particularly their genome encapsulation. Throughout evolution, viruses have developed various strategies to protect their genomes from destruction by host defense mechanisms, leading to large structural diversity in single-stranded RNA (ssRNA) encapsulation (1). Nevertheless, there are some common principles for ribonucleoprotein (RNP) assembly in members of the *Bunyavirales* order, an order of single-stranded, segmented, negative-sense RNA viruses.

First described in 1933, fig mosaic disease (FMD) is a cosmopolitan disorder of Ficus carica (common fig) that is characterized by multiple symptoms, especially on tree foliage, including chlorotic mottling, vein clearing and banding, ring spots, and small mottled fruits (2). Only recently was it shown that the fig mosaic emaravirus (FMV) was the causal agent of FMD (3). Eriophyid mites (Acaria ficus) are the transmitting vector of FMV (4, 5) that infect almost all fig orchards worldwide. However, several studies showed that the plant host range of FMV appears to be extended outside the *Moraceae* family (4, 6).

As an emaravirus belonging to the *Fimoviridae* family (order *Bunyavirales*), FMV shares a similar morphology with other emaraviruses, including the European mountain ash ringspot-associated virus (7). Emaraviruses are enveloped viruses 80 to 100 nm in diameter with a multipartite, negative-sense, single-stranded RNA genome that is encapsulated by the nucleocapsid (N) protein (8).

Canonically, FMV contains four ssRNA segments, RNA1 through RNA4, encoding the RNA-dependent RNA polymerase (RdRp), the glycoprotein precursor, the nucleoprotein (N), and a movement protein, respectively (3, 9). Recent reports described two additional genomic segments for FMV (RNA5 and -6) with unknown open reading frames (ORFs) (10, 11). Little is known about the mechanism of viral genome encapsulation in emaraviruses and the role of N proteins in the virus infection cycle in mites and plants. However, it has been shown that the N proteins form agglomerates in the cytoplasm that are predominantly enveloped by the endoplasmic reticulum (ER) membrane.

N proteins from members of the order *Bunyavirales* vary in molecular weight, ranging from 27 to 57 kDa (12–14). Interestingly, although one might expect a significant resemblance of the N protein structures within phylogenetically related viruses, there is a weak sequence similarity of N proteins from different families in the *Bunyavirales* order. The available crystal structures show highly similar, family-specific characteristics that might be derived from the vast diversity of vectors and hosts infected by different bunyaviruses (15).

In general, N proteins of bunyaviruses consist of a globular core that includes the RNA binding cleft and two terminal arms (N and C arms) that enable oligomerization. In contrast, the N proteins of orthonairoviruses adopt a head and stalk architecture (13), where the head domain has structural homology to N from arenaviruses (family *Arenaviridae*), a negative-sense ssRNA virus family that only recently was included in the *Bunyavirales* order (16). Previously, X-ray crystallography experiments demonstrated that nucleoproteins of various bunyaviruses not only assemble in several oligomerization states but can also form filamentous assemblies in the crystal packing (13, 14, 17–19). In accordance with that, a recent cryo-electron microscopy (cryo-EM) structure of hantavirus (family *Hantaviridae*) N bound to ssRNA showed a helical filament formation of N upon RNA binding (20).

As yet, there is no structural information on genome encapsulation in emaraviruses, particularly in FMV. The oligomeric states of their nucleoproteins and the mode of RNA binding are yet to be determined. Here, we present the crystal structures of FMV nucleoprotein in its RNA-free form and in a complex with a single-stranded RNA fragment.

## RESULTS

### Expression, purification, and characterization of recombinant FMV N.

We subcloned the cDNA encoding the fig mosaic virus nucleoprotein (FMV N; GenBank accession number NC_029563) in-frame with a removable N-terminal 6×His tag into a pET28 vector for bacterial expression. We expressed full-length N protein (N^fl^) in Escherichia coli cells and purified it in its RNA-free form using chromatographic methods (Fig. S1A in the supplemental material). During the first immobilized-metal affinity chromatography (IMAC) step of purification of N^fl^, we noticed that the expressed protein was copurified with what seemed to be bacterial RNA, as the *A*_260_/*A*_280_ absorbance ratio was ~1.5. An additional wash of the IMAC resin with 3 M urea prior to elution removed the nucleic acid contamination. Preparative size exclusion chromatography (SEC) showed that N^fl^ eluted in two distinct peaks: a void-volume peak, representing a high-molecular-weight N^fl^ in complex with remnants of the bacterial RNA (as evaluated by an *A*_260_/*A*_280_ ratio of 1), and an RNA-free form of N^fl^ (*A*_260_/*A*_280_ of ~0.5), representing an oligomeric form of the protein (Fig. S1A). A size exclusion chromatography with multiangle light scattering (SEC-MALS) experiment exhibited a prominent peak that consisted almost entirely of tetramers, though trimer and pentamer populations were also observed to a much lesser extent (Fig. S1B).

### Structure of RNA-free FMV N.

**FIG 1:**
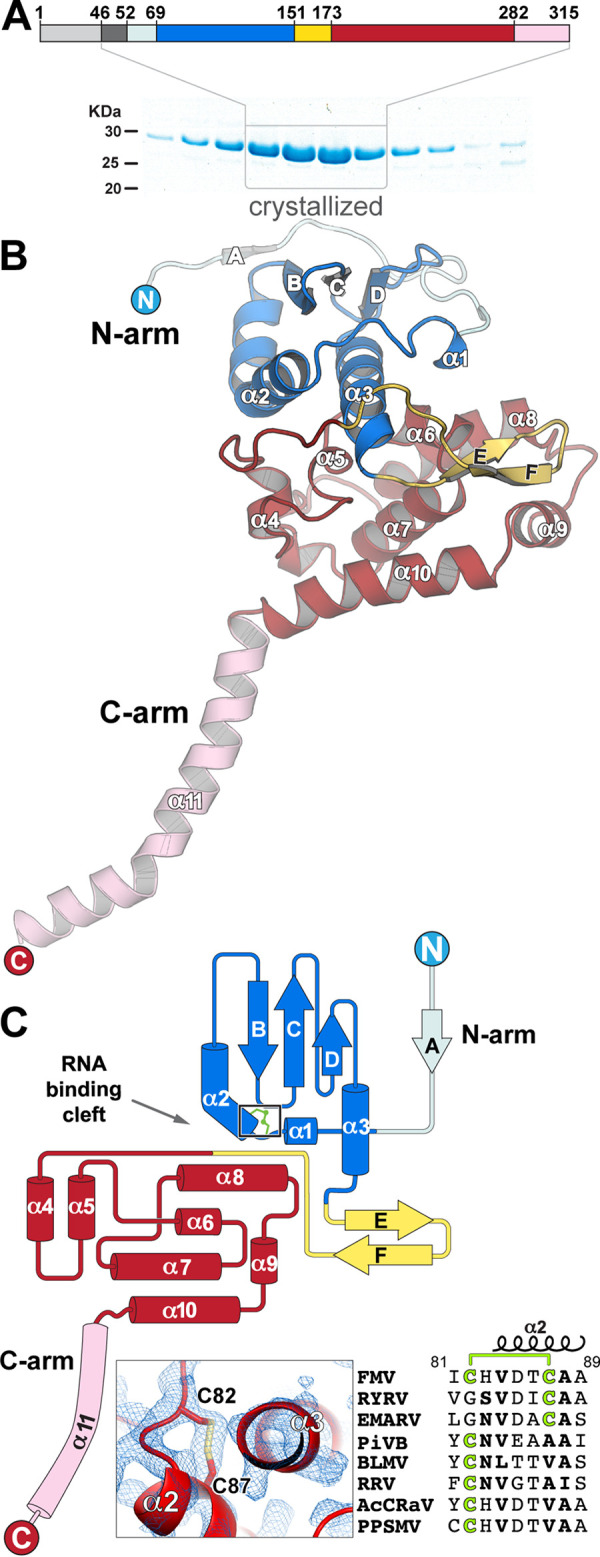
FMV N structure overview. (A) A linear representation of FMV N’s domain organization with a representative SDS-PAGE analysis of the fractions from the SEC, the final purification step. Light gray, N-terminal region removed with trypsin treatment; dark gray, the disordered region that was not visible in the electron density map; light blue, N-terminal arm; blue, N-terminal lobe; yellow, EF hairpin insertion; red, C-terminal lobe; pink, C-terminal arm. (B) Cartoon representation of FMV N monomer with nomenclature assignments of secondary structures. (C) Topology diagram of FMV N. In the inset, a 2F_o_-F_c_ electron density map at 1.2 σ shows the intramolecular disulfide bond between C82 and C87.

Since the RNA-free N^fl^ failed to crystallize in a reproducible manner, we used limited-proteolysis analysis, utilizing various proteases to determine the domain composition and boundaries of FMV N (Fig. S2). Only the trypsin-treated sample showed a homogeneous proteolytic product. Mass spectrometry combined with N-terminal sequencing analysis of the trypsin-treated N confirmed the elimination of the first 45 N-terminal residues (Fig. 1A). The trypsin-treated N protein (N^ΔN45^) was then purified and screened for crystallization conditions. Once the crystallization conditions were optimized, we used a single isomorphous replacement with anomalous signal (SIRAS) experiment to calculate phases to 3 Å, and an initial structure was built manually. Finally, the structure was refined against a 2.28-Å-resolution data set with excellent statistics (Table 1).

**TABLE 1:** Crystallographic data collection and refinement statistics

Parameter	N^ΔN45^ (SIRAS nat)	N^ΔN45^ (SIRAS Hg)	N^ΔN45^ (high resolution)	N^ΔN45^-42mer complex
PDB ID			8AX4	8AXF
Data collection statistics				
Wavelength (λ)	1.04	1.009	1.072	1.072
Resolution range (Å)^*a*^	49.46–3.0 (3.107–3.0)	49.46–2.95 (3.055–2.95)	48.46–2.28 (2.34–2.28)	48.12–2.542 (2.633–2.542)
Space group	P2_1_	P2_1_	P2_1_	P2_1_2_1_ 2_1_
Unit cell lengths (Å)	100.286 96.124 103.182	100.286 96.124 103.182	99.87 96.92 103.52	53.17 151.82 169.57
Unit cell angles (°)	90 110.9 90	90 110.9 90	90 111.9 90	90 90 90
Total reflections	244,170 (24,042)	258,611 (26,567)	296,115 (21,934)	335,800 (28,832)
Unique reflections	36,514 (3,640)	38,679 (3,815)	82,002 (6,021)	45,869 (4,160)
Multiplicity	6.7 (7.1)	6.7 (6.9)	3.6 (3.6)	7.3 (6.9)
Completeness (%)	95 (93)	94 (100)	99 (99.0)	99.19 (92.59)
Mean *I*/σ(*I*)	11.96 (1.75)	10.64 (1.54)	15.31 (0.94)	13.34 (1.27)
Wilson B factor (Å^2^)	96.93	94.76	38.45	69.49
*R*_merge_ (%)^*b*^	0.07438 (0.8839)	0.09461 (1.182)	0.067 (1.61)	0.12 (1.52)
*R*_meas_ (%)	0.08082 (0.9537)	0.1028 (1.277)	0.079 (1.89)	0.1299 (1.641)
CC_1/2_	0.999 (0.912)	0.998 (0.786)	0.99 (0.51)	0.997 (0.53)
CC*	1 (0.977)	1 (0.938)	1 (0.9)	0.999 (0.832)
Refinement statistics				
Reflections used in refinement			79,762 (606)	45,824 (4,160)
Reflections used for *R*_free_			1,997 (18)	2,000 (182)
*R*_work_^*c*^			0.2065 (0.3729)	0.2265 (0.3887)
*R*_free_^*c*^			0.1983 (0.4055)	0.2447 (0.4090)
CC_work_			0.949 (0.689)	0.951 (0.676)
CC_free_			0.947 (0.583)	0.949 (0.556)
No. of: Nonhydrogen atoms			9,051	9,387
Macromolecules atoms			8,282	9,093
Ligand atoms			8	9
Protein residues			1,039	1,039
RMSD (bonds) (Å)^*d*^			0.009	0.006
RMSD (angles) (°)^*d*^			0.99	0.96
Ramachandran plot				
Favored regions (%)			97	97.86
Allowed regions (%)			2.8	2.14
Outlier regions (%)			0	0
Rotamer outliers (%)			0.86	4.87
Clashscore			5.1	6.21
Avg B factor (Å)			50.53	82.2
Macromolecules			50.23	81.94
Ligands			57.62	100.87
Solvent			53.69	83.97

Beamline	BESSY 14.1	DESY P13	BESSY 14.1	ESRF ID23-1

a Highest-resolution shell is shown in parentheses.

b *R*_merge_ = Σ*_hkl_Σ_i_*|I*_hkl_*_,_*_i_ – _hkl_|/Σ_hkl_Σ_i_|I_hkl_*_,_*_i_|*, where *I_hkl_* is the intensity of a reflection and *_hkl_* is the average of all observations of the reflection.

c *R*_free_, *R*_work_ with 10% of *F*_obs_ sequestered before refinement.

d RMS, root mean square deviation. SIRAS - Single Isomorphous Replacement with Anomalous Signal, nat - native crystal, Hg - Mercury derivetized crystal.

The crystal structure of RNA-free N^ΔN45^ revealed four N^ΔN45^ molecules in the asymmetric unit that were organized in a tight tetramer, as was previously reported for N proteins from other members of the *Bunyavirales* order (14, 18, 21, 22). Each of the monomers comprised an N-terminal arm (N arm, residues 52 to 71), a globular core (residues 71 to 280), and an α-helical C-terminal arm (C arm, residues 281 to 313) (Fig. 1B). Residues 46 through 51 were not visible in the electron density map, and therefore, it is plausible that they were disordered. The N arm was mostly a coiled loop that contained a short β-strand (Fig. 1B and C, strand A), followed by the N-terminal subdomain (N lobe) of the globular core, which encompassed three α-helices (α1 to α3), and the BCD β-sheet, which was inserted between α2 and α3 (Fig. 1B). The C-terminal subdomain of the core (C lobe) consisted of helices α4 through α10 and was followed by the C arm, a 33-residue-long amphipathic α-helix (Fig. 1, Fig. S3). The putative RNA binding cleft, a positively charged trench, was formed between the N and C lobes, and as expected, it did not contain any RNA molecules. Interestingly, unlike other bunyaviruses’ nucleoproteins, the FMV N presented one disulfide bond between C82 and C87 that was observed in all four copies of the asymmetric unit (Fig. 1C, bottom). Multiple-sequence alignment revealed that other emaraviruses possessed a cysteine residue in either of the equivalent positions but not in both (Fig. 1C, Fig. S4), implying that this disulfide bond might not be essential for the protein’s function.

**FIG 2:**
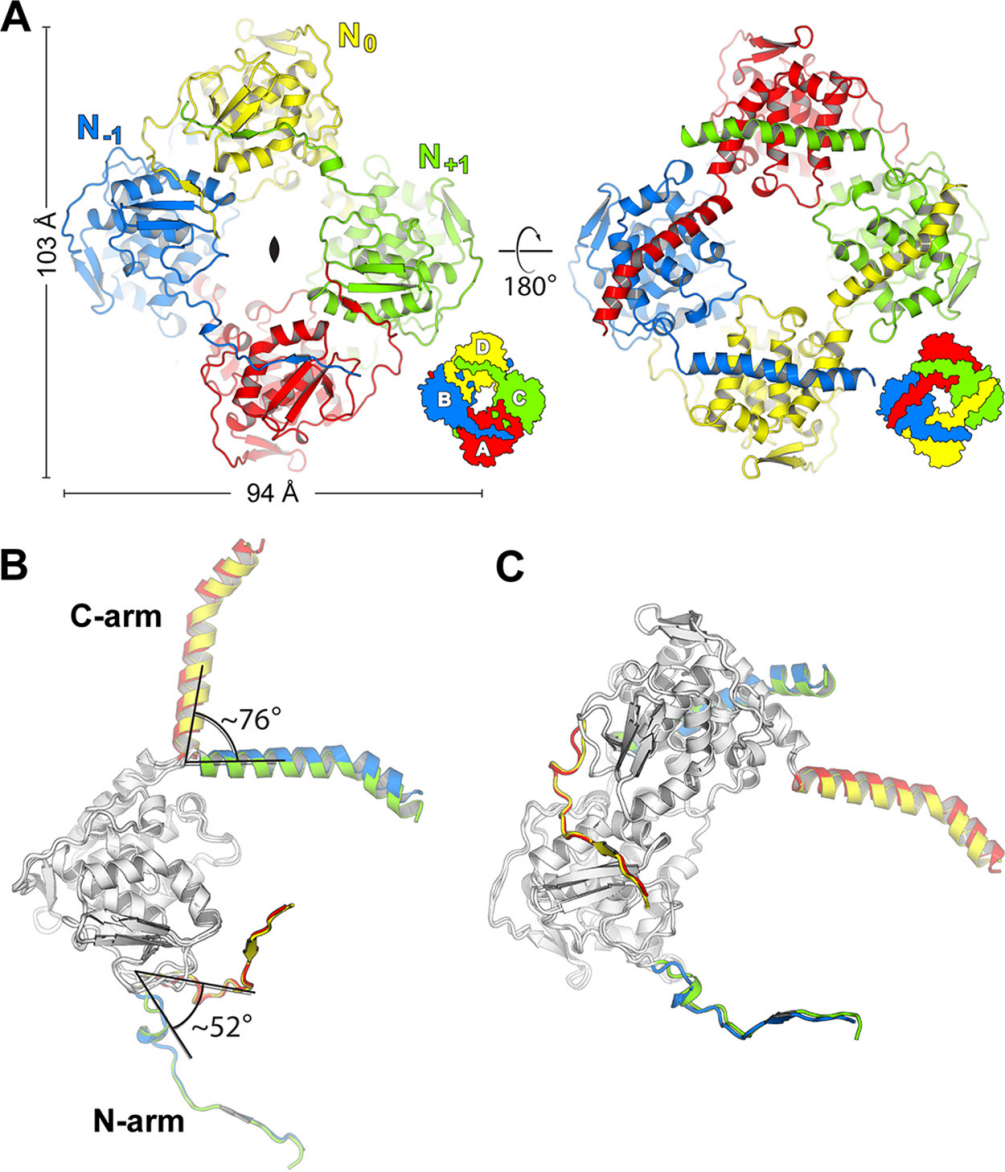
Organization of the apo-FMV N tetramer. (A) Top view (left) and bottom view (right) of the FMV N tetramer. (B) Superposition of all protomers in the tetramer assembly of FMV N. The protomers can be divided into two conformers. The globular core is light gray, whereas the N- and C-terminal arms are colored according to the protomer colors in panel A. (C) Superposition of AB dimers (red and blue) onto the CD dimer (green and yellow). The FMV N tetramer is a dimer of two identical, asymmetric dimers.

The FMV N tetramer was organized as two dimers with 2-fold symmetry rather than having a 4-fold symmetry (Fig. 2A). The protomers could be classified into two types that differed in their N and C arm orientations with respect to the core (Fig. 2B); however, no major differences in the core domains were observed between the two protomer types. This organization gave rise to two identical dimers (Fig. 2C, A-B and C-D dimers) that assembled into the tetramer. A similar asymmetric tetramer organization of the nucleoprotein was previously reported for members of the *Peribunyaviridae* family, which are animal-infecting bunyaviruses (23–25). Each protomer (N_0_) interacted with two other protomers via its N arm, which interacted with the N_−1_ protomer, and its C arm, which interacted with the N_+1_ protomer (Fig. 2A). Strand A at the N arm of N_0_ interacted with the B strand at the BCD β-sheet and was reinforced by additional polar interactions. In contrast, the interactions of the C arm with the N_+1_ protomer were primarily hydrophobic and mediated mainly by the conserved residues A290, F294, L297, and F301 (Fig. S3 and S4). Markedly, the interface between the C arm and the N_+1_ protomer was significantly more conserved than the N arm’s interface with the N_−1_ protomer (Fig. S3).

### FMV N-RNA interaction.

**FIG 3:**
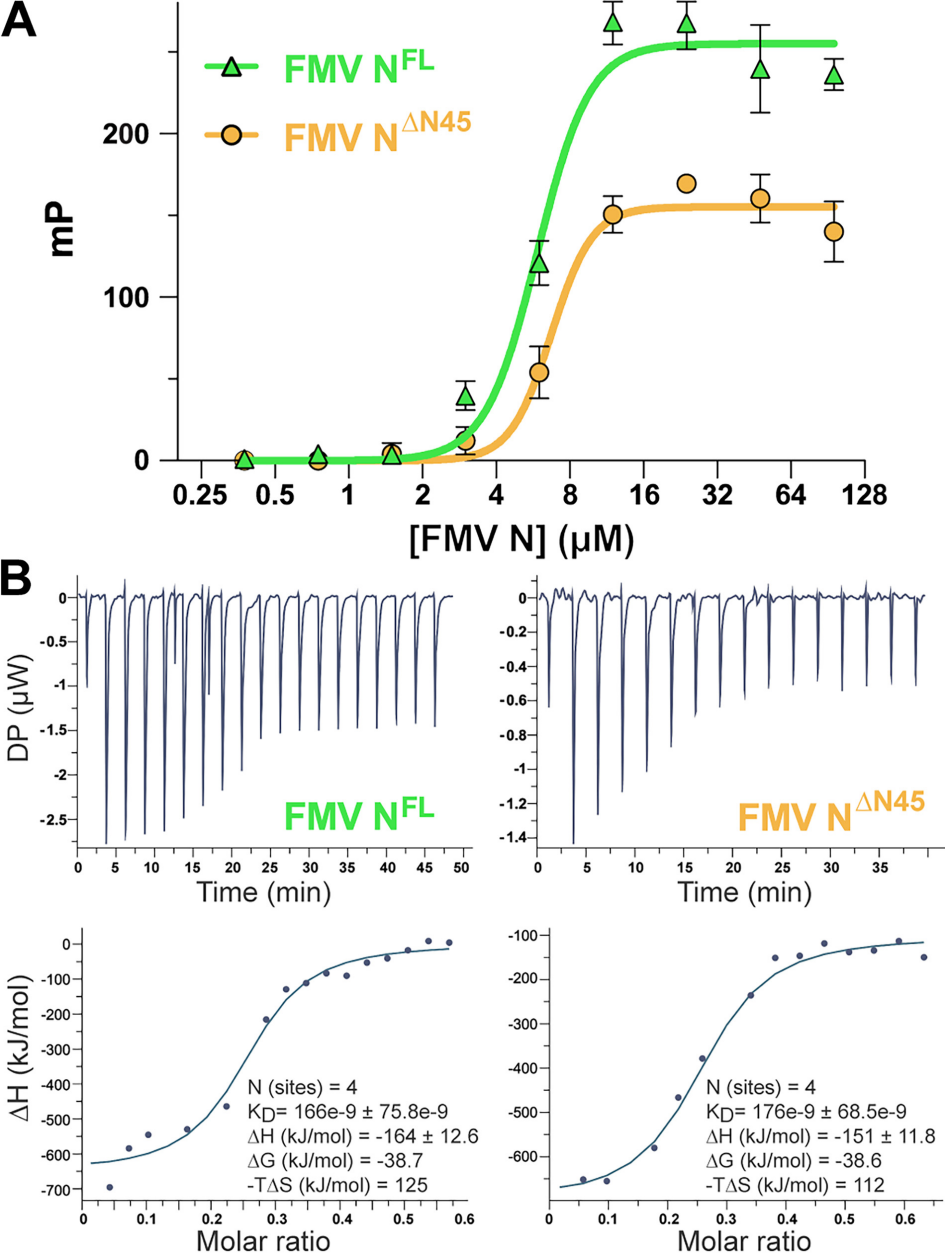
FMV N binds to a 42-nt ssRNA fragment. (A) Fluorescence polarization measurements of the interaction of N^fl^ (green) and N^ΔN45^ (orange) with fluorescently labeled (5′) 42-nt poly(U) RNA. The calculated *K_D_* values are 5 and 6.6 μM, respectively. Error bars show standard deviations. mP, polarization units calculated from the perpendicular and parallel fluorescence intensity values relative to the direction of the polarized excitation light. (B) Isothermal calorimetry (ITC) measurements of unlabeled 42-nt poly(U) RNA. *K_D_* and thermodynamic values are shown. DP, differential power.

To evaluate the RNA binding properties of the purified FMV N^fl^ and N^ΔN45^, we tested their RNA binding characteristics to a short, 42-nucleotide (nt)-long polyribouridylate oligoribonucleotide [poly(rU)] that was previously used to characterize the encapsulation properties of other N proteins from members of the *Bunyavirales* order (26). Using fluorescence polarization (FP) measurements with a 5′ fluorescently labeled 42-mer, we calculated the binding constant of RNA to either N^fl^ or N^ΔN45^ to *K_D_* (equilibrium dissociation constant) values of ~5 μM and 6.6 μM, respectively (Fig. 3A). However, it was reported previously that N proteins from other bunyaviruses bind RNA with *K_D_* values in the low-nanomolar range (21, 27). Thus, we conducted isothermal calorimetry (ITC) experiments to measure the binding of the label-free, 42-nt-long ssRNA poly(rU) to purified N tetramers (Fig. 3B). These measurements resulted in a *K_D_* of ~70 nM, similar to the *K_D_* values of nucleoproteins from other bunyaviruses (21, 27). We reasoned that these measurement discrepancies were due to the presence of the 5′ fluorophore, which might sterically interfere with the binding of the oligonucleotide’s 5′ end.

### Structure of FMV N^ΔN45^ in complex with RNA.

**FIG 4:**
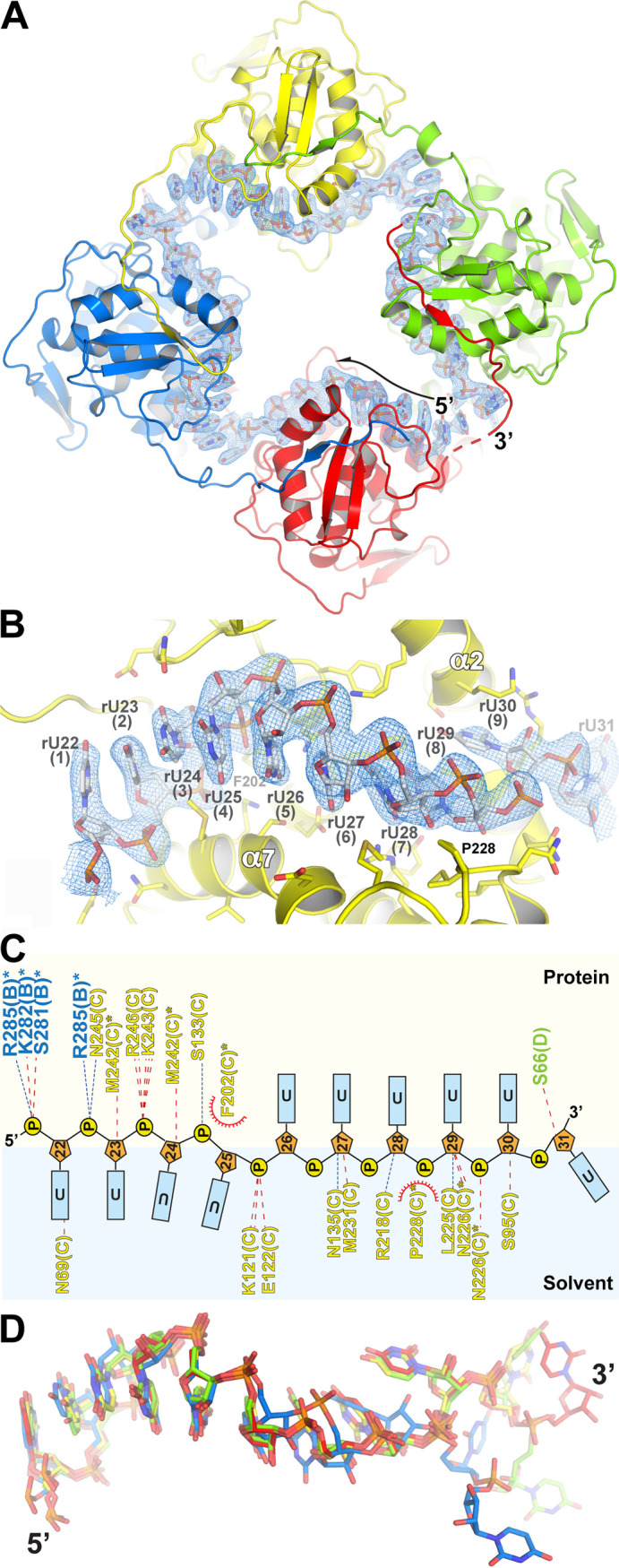
Crystal structure of FMV N in complex with 42-nt ssRNA fragment. (A) Top view of the FMV tetramer-RNA complex. The N protomers and RNA are shown in a cartoon and stick representation, respectively. The blue mesh is a 2F_o_-F_c_ electron density map at 1.5 σ that masks the RNA. (B) Magnified view of the RNA binding cleft of an N protomer. The interacting residues are shown in stick representation. (C) Schematic plot of the N-RNA interaction. The interacting residues are colored according to the protomer (blue, B; yellow, C; green, D). The light tan background (top) represents the protein side, and the light blue background (bottom) represents the solvent. (D) Superposition of the RNAs from all four protomers within a tetramer. Note that the RNA binding conformation in all protomers is conserved at the 5′ end and diverges toward the 3′ end.

To explore the RNA encapsulation mode of FMV N, we cocrystallized the FMV N tetramer with the 42-mer poly(rU) and obtained crystals that belonged to the P2_1_2_1_2_1_ space group with unit cell dimensions of *a *= 53.168 Å, *b* =151.823 Å, *c *= 169.575 Å, α = β = γ = 90°. The structure was determined using molecular replacement with the RNA-free monomer’s core as the search model. The N- and C-terminal arms and all 42 nucleotides were resolved in the electron density map, and the entire protein and RNA molecules were built manually. Finally, the structure was refined to 2.5-Å resolution (Table 1, Fig. 4).

The structure of FMV’s N-RNA complex presents a tetrameric ring that forms a continuous positively charged groove in its inner diameter, where the RNA molecule is threaded (Fig. 4A). This RNA binding groove is enriched with positively charged amino acids, mainly lysine and arginine residues. Each protomer covers nine nucleotides; four bases face the solvent, and five nucleotides have their bases facing the protein (Fig. 4B and C). The latter occupy the RNA binding cleft formed by helix α2, the linker between β-strands C and D (CD loop), α3 from the N-terminal lobe (NTL), and α4, α7, and α8 from the C-terminal lobe (CTL) (Fig. 4B and C). In each protomer, the 5′ base 1 is interacting with N69 and the RNA is held in a pseudohelical conformation enforced by van der Waals interactions of F202 and P228 with the RNA’s backbone (Fig. 4B). Between every two protomers of N, there is a two-nucleotide linker that forms a sharp turn in the RNA backbone, which allows the successive nine nucleotides into the RNA binding cleft of the next protomer. In general, all protomers bind the RNA molecule in the same manner. Nevertheless, there are structural divergences in the orientation of the nucleotide at the 3′ end, as the RNA leaves the N_0_ protomer and continues to the N_+1_ protomer (Fig. 4D).

### The N tetramer rearranges upon RNA binding.

**FIG 5:**
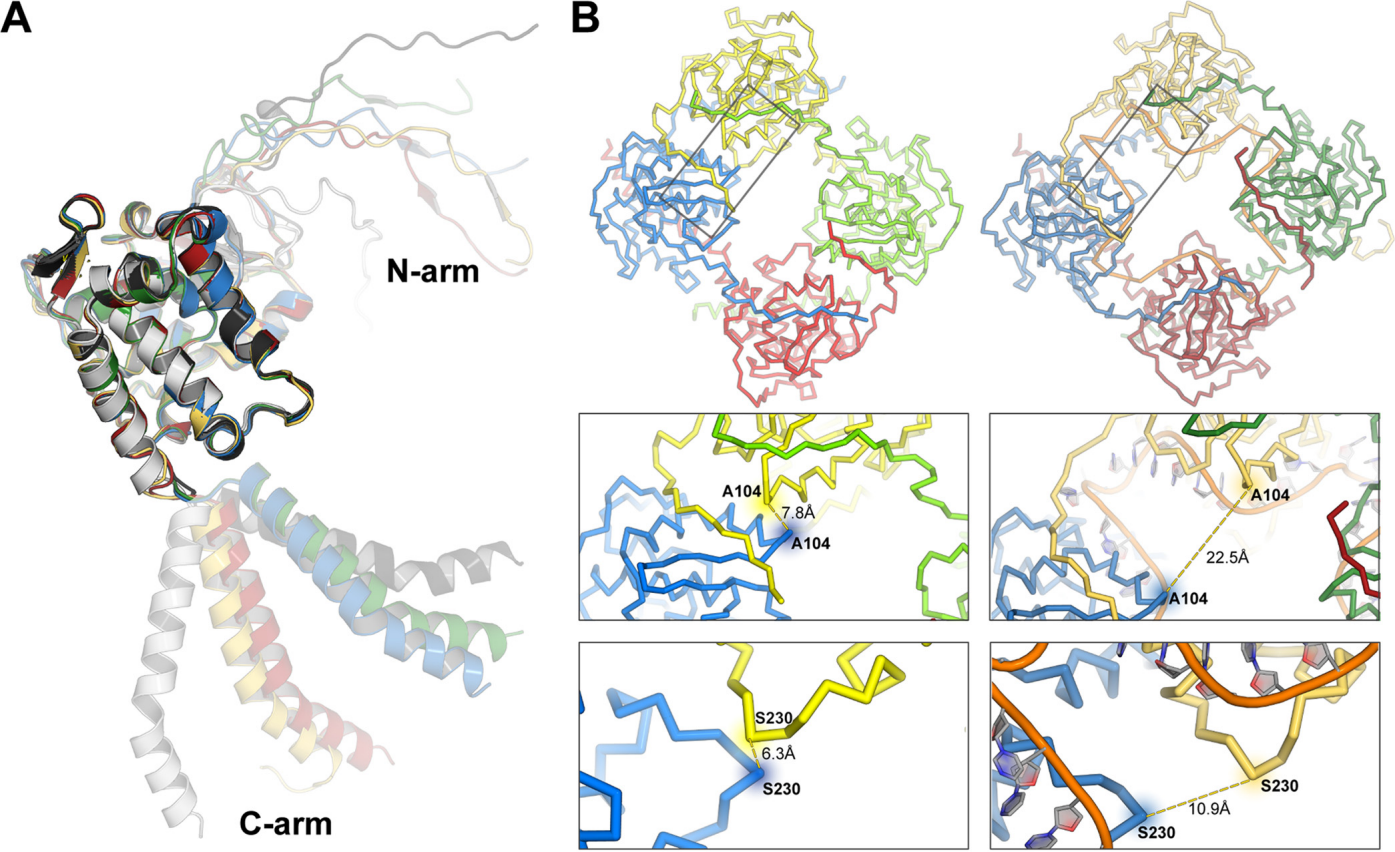
The FMV N tetramer changes conformation upon binding to RNA. (A) Superposition of the protomers in the N-RNA complex (colors) onto the apo-NP protomers (shades of gray), with the globular core as the reference set of atoms. (B) Comparison between the tetramer organization of the RNA-free and RNA-bound N tetramers. The magnifications show representative differences between the two structures.

Although it resembles the RNA-free form of the N tetramer, the N-RNA complex reveals a displacement of the N protomers with respect to each other and changes in the orientation of the two terminal arms in respect to the subunit’s core (Fig. 5). In contrast, there are no significant structural alterations in the globular core domain and the residues of the RNA binding cleft. Upon binding to the RNA, the tetramer adopts an open conformation by utilizing a “paper fortune teller”-like transformation (Movie S1). The major displacements occur between the two sets of dimers (dimers A-B and C-D) as the two dimers move apart so that the RNA can be accommodated (Fig. 5B). This alteration of the N tetramer organization is what allows the RNA molecule to bind. A superposition of the RNA-free N tetramer onto the RNA-bound structure shows major clashes between N and the RNA in all four protomers (Fig. S5), which implies that a conformational change of the tetramer must occur for the RNA to bind. Finally, there is a weak electron density of the linker between the N arm and the globular core, suggesting a flexible region that allows a range of distances between the two dimers in the tetramer.

## DISCUSSION

The ribonucleoprotein (RNP) complex of single-stranded RNA viruses is formed by encapsulating the viral genomic RNA with multiple copies of the nucleoprotein. The N protein serves as the main building block for the RNP assembly, protects the viral genome from endo- and exonucleases, and helps to evade the host’s innate immune response. Although they have the same function, there is little sequence similarity between N proteins of different members of the *Bunyavirales* order (25, 28).

Here, we describe the crystal structure of FMV N in its RNA-free form and in complex with a 42-nt fragment [poly(rU)]. FMV N is found exclusively as an asymmetric tetramer within the crystals. The tetrameric form is also the prevalent oligomeric state of N in solution, as determined by SEC-MALS (Fig. S1B). Such asymmetric tetramer organization was previously observed in the crystal structures of N proteins from members of the *Peribunyaviridae* virus family (14, 18, 19, 21). Our two structures, the apo-N and the N-ssRNA complex, provide a clear explanation of the genome protection offered by FMV N.

**FIG 6:**
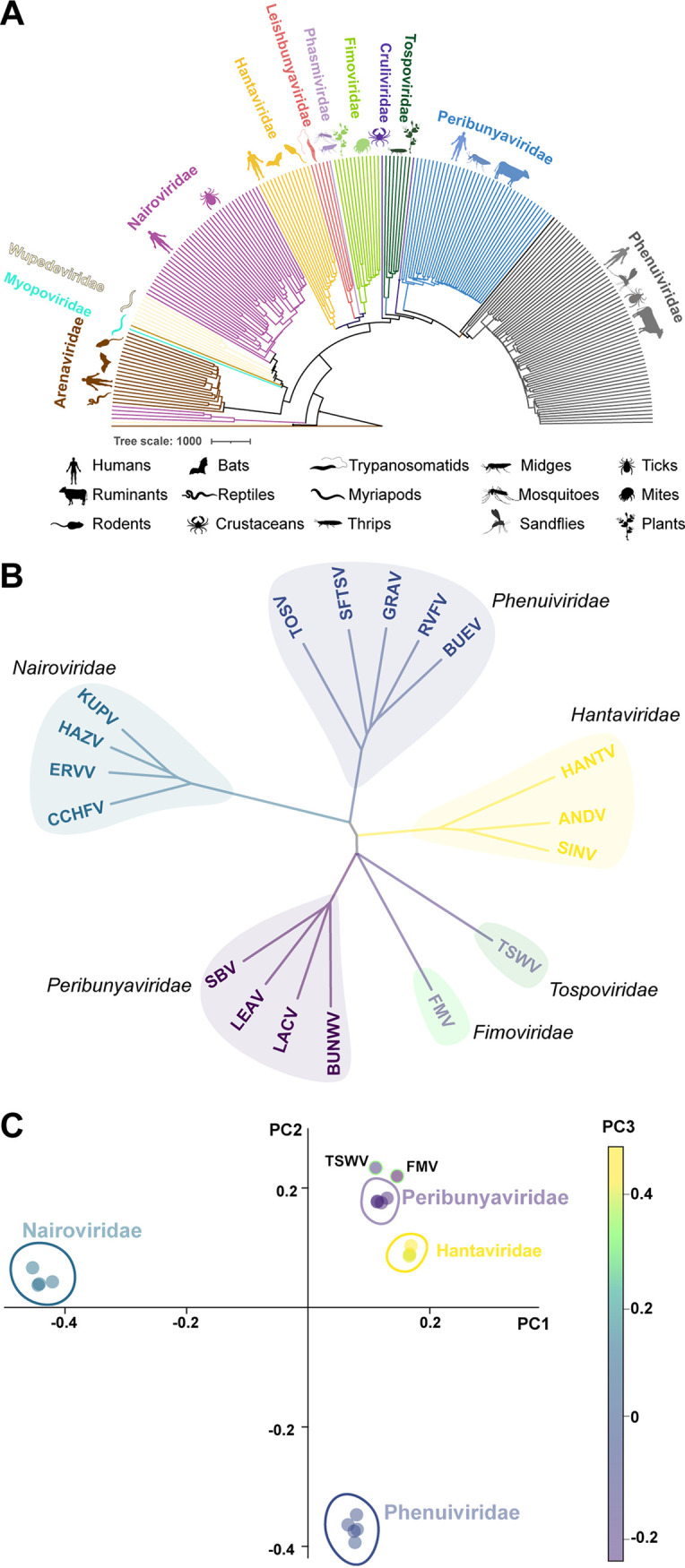
Evolutionary analysis of FMV N. (A) Sequence-based phylogenetic tree of N proteins from the *Bunyavirales* order. Each color represents a family, and icons represent hosts as shown in the key. (B) Phylogenetic tree calculated using structure-based sequence alignment of the available bunyavirus N structures (PDB IDs 4J1J, 4JNG, 4IJS, 5E04, 5E06, 5FSG, 4H5P, 4J4W, 4J4X, 4J4R, 5A97, 4XZC, 4XZ8, 4AQG, 5FVA, 4BGP, and 5Y6J). TOSV, Toscana virus; SFTSV, severe fever with thrombocytopenia syndrome virus; GRAV, Granada virus; RVFV, Rift Valley fever virus; BUEV, Buenaventura virus; HANTV, hantavirus; ANDV, Andes virus; SINV, Sin Nombre virus; TSWV, tomato spotted wilt virus; FMV, fig mosaic virus; BUNWV, Bunyamwera virus; LACV, La Crosse virus; LEAV, Leanyer virus; SBV, Schmallenberg virus; CCHFV, Crimean-Congo hemorrhagic fever virus; ERVV, Erve virus; HAZV, Hazara virus; KUPV, Kupe virus. (C) Principal-component analysis (PCA) of the tree in panel B.

The structure of FMV N encompasses the same structural elements as N proteins from other bunyaviruses: a globular core that comprises two lobes and forms a positively charged cleft where the viral RNA is threaded (Fig. S6). Despite the low sequence homology and the structural differences, the organization of the secondary structures around the RNA binding cleft of FMV N resembles that of the secondary structures around the RNA binding clefts of both peribunyaviruses and tospoviruses (Fig. S6B). This structural resemblance implies that N proteins from members of the *Fimoviridae*, *Tospoviridae*, and *Peribunyaviridae* families share an ancestor. To further investigate the evolutionary link between plant- and animal-infecting bunyaviruses, we modeled a sequence-based phylogenetic tree of *Bunyavirales* N proteins (Fig. 6A). The two plant-infecting bunyaviruses (fimoviruses and tospoviruses) are each classified in a different clade that encompasses different animal-infecting viruses (*Peribunyaviridae* and *Phasmaviridae*, respectively). However, a dendrogram we generated using a structure-based sequence alignment shows that N proteins from plant-infecting bunyaviruses have a common ancestor with peribunyaviruses (Fig. 6B). A principal-component analysis of the phylogenetic tree indicates that N proteins from peribunyaviruses and N proteins of plant-infecting bunyaviruses are clustered and depart significantly from other N proteins from animal-infecting bunyaviruses (Fig. 6C). The resemblance of the FMV N tetramer assembly to those of peribunyavirus N proteins and the structure-based sequence alignment imply that plant-infecting bunyaviruses and peribunyaviruses share an ancestor.

### The mode of assembly of FMV RNP.

**FIG 7:**
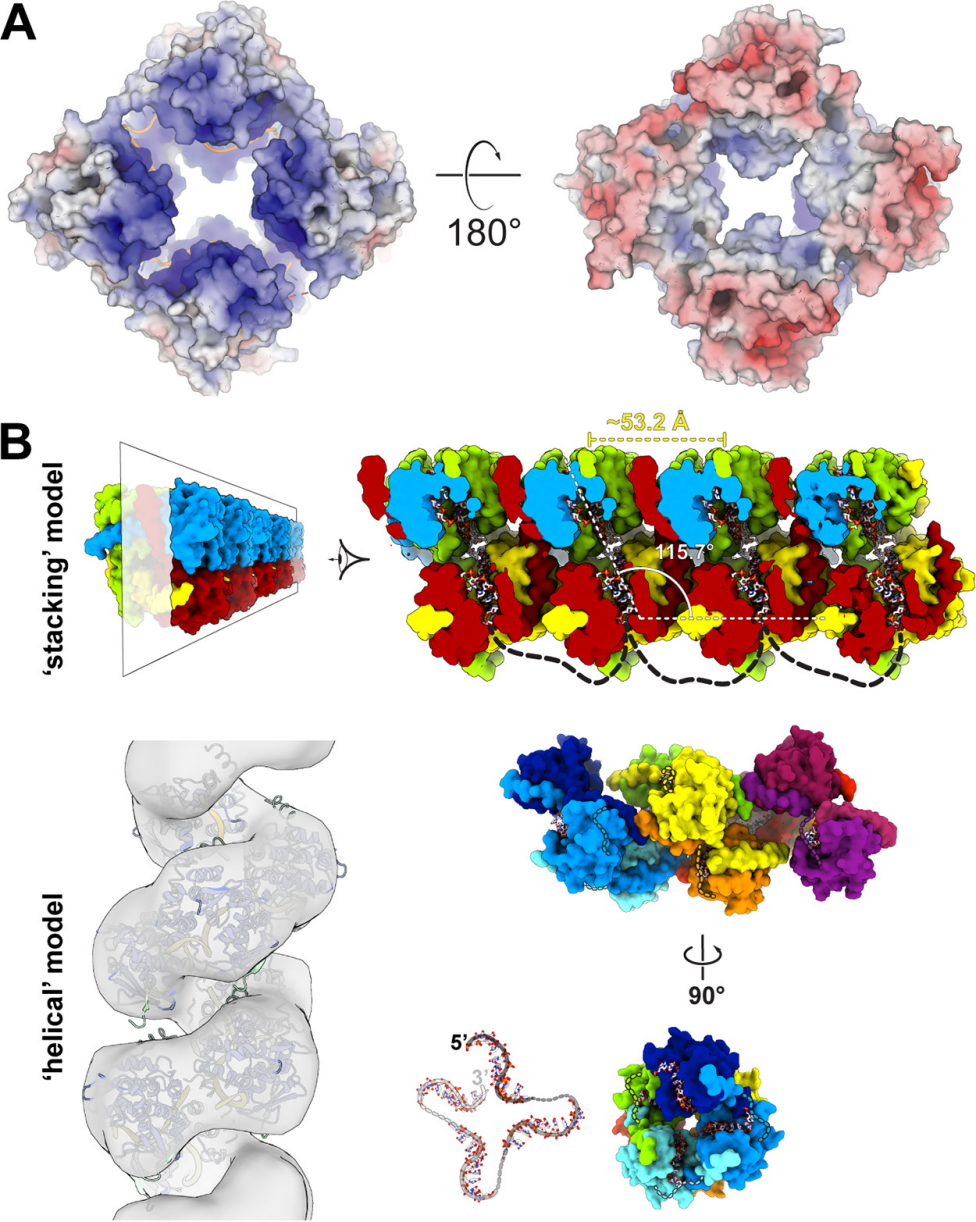
Assembly model for FMV ribonucleoprotein (RNP) complex. (A) Surface electrostatic potential of FMV N tetramer. Top (left) and bottom (right) views of N tetramer in complex with ssRNA in a surface representation colored by the surface electrostatic potential calculated by APBS (https://server.poissonboltzmann.org/). (B) Two possible models of FMV N. The stacking model is supported by the filamentous crystal packing of the tetramer of FMV N-ssRNA. The cross-section demonstrates the RNA (stick representation) packed within the tetramer. The helical model is supported by the cryo-EM reconstruction of the Bunyamwera virus (BUNV) native RNP. Using UCSF Chimera, FMV N was fitted into the 13-Å-resolution volume (EMD_11847), resulting in model of helical assembly of FMV N. In the center bottom is a view through the helical axis of the RNA trajectory in the helical model. The dashed lines in gray shades represent the missing nucleotides between the 10 nucleotides that interact with each copy of N. Dark gray, front; light gray, back.

FMV N was shown to bind the 5′-terminal sequence of FMV’s RNA5 segment (280 nt) with no sequence specificity (29), but the mode of assembly of FMV N, as well as that of other bunyaviruses, on a long ssRNA fragment is still unclear. The structural similarities between FMV N and N proteins from peribunyaviruses suggest that the RNP assembles similarly in both families. Previously it was reported that different crystal structures of N from La Crosse virus (LACV; family *Peribunyaviridae*) contained monomers or tetramers in the asymmetric unit and were also found to form a helical assembly throughout the crystal (19). Electrostatic surface potential analysis of the FMV N tetramer shows that the two faces of the tetramer have substantial complementary charges (Fig. 7A), and the crystal structure of the N-ssRNA complex shows that the N tetramers exhibit a filamentous arrangement throughout the crystal where the tetramer plane is at an ~116° angle with respect to the filament axis (Fig. 7B, top). This filament-like structure is favorable, possibly due to the charge complementarity between the two faces of the tetramers. Therefore, one can suggest that the building block of FMV’s RNP is a tetrameric form of N in which each tetramer binds to ~42 nucleotides. This kind of assembly will be rigid and might not allow efficient assembly of the viral genome into spherical virions. Therefore, an RNA linker should be present between tetramers to enable flexibility.

As of now, few EM-based studies of selected bunyaviruses demonstrate that native RNPs isolated from virions exhibit a linear morphology with a diameter greater than that of a monomer, suggesting that a monomeric N might not be the RNP’s building block (12, 18, 27, 30, 31). However, as was shown for La Crosse virus (LACV; family *Peribunyaviridae*), the native RNP can adopt a highly ordered helical assembly, as observed in the crystal lattice of the recombinant LACV N (19). Recently, a pseudoatomic model of a native RNP complex from Bunyamwera virus (BUNV; family *Peribunyaviridae*) was released, based on cryo-electron microscopy (cryo-EM) with an average 13-Å resolution (32). The calculated volume demonstrates a relaxed helical architecture with an ~18-Å pitch. Our evolutionary analysis shows that N proteins from fimoviruses and peribunyaviruses might have a common ancestor, and therefore, it is plausible that their RNP assemblies will be similar. To fit FMV N to the cryo-EM volume of BUNV RNP, we generated a composited building block comprised of the core of N_0_ with 10 nucleotides bound to it, the N-terminal arm from N_+1_, and the C-terminal arm from the N_−1_ protomer (Fig. 2A). We then superimposed these coordinates onto the pseudoatomic model of BUNV RNP (Fig. 7B) to generate a helical model of FMV RNP. Intriguingly, not only are the termini of the gapped region at a distance that can accommodate the missing residues, but the projection of the RNA throughout the helical assembly resembles the shape of the RNA bound to the tetramer (Fig. 7B, Fig. S7A). Indeed, the electrostatic surface potential can also support the helical model; however, experimental evidence is required to determine the mode of assembly of the FMV RNP.

In conclusion, in this work, we determined the crystal structures of FMV N in its RNA-free form and in complex with a 42-nucleotide ssRNA fragment. The FMV N fold and oligomerization mode suggest an evolutionary link between plant-infecting viruses and animal-infecting viruses from the *Peribunyaviridae* family. Using our structural approach, complemented by biochemical and biophysical measurements, we have characterized the N-ssRNA interactions and hypothesized two models for the FMV RNP assembly. It will be of interest to the field to elucidate a high-resolution structure of either native RNP or the assembly of N on a longer ssRNA fragment to understand the genome packing of members of the *Fimoviridae* virus family.

## MATERIALS AND METHODS

### Protein expression and purification.

The full-length FMV N gene (N^fl^, isolate SB2-4, GenBank accession number AB697854.1) was codon optimized for E. coli expression and synthesized at BioBasic. The synthetic product was subcloned into the pET28a expression vector between the NdeI and NotI restriction sites in frame with an N-terminal 6× histidine purification tag and a tobacco etch virus (TEV) protease cleavage site. pET28a-FMV N^fl^ was transformed into E. coli Rosetta(DE3) strain cells (Novagen). A single colony was used to inoculate 50 mL of LB medium supplemented with kanamycin and chloramphenicol antibiotics (LB^Kan+Cam^). The culture was incubated with shaking at 37°C, 200 rpm, for 16 h. This starter culture was then used to inoculate 1.5 L LB^Kan+Cam^ at 37°C, 200 rpm, until it reached an optical density at 600 nm (OD_600_) of 0.6 to 0.8, when the expression was induced with 1 mM IPTG (isopropyl β-d-1-thiogalactopyranoside) at 20°C, 200 rpm, for 14 h. The cells were harvested (10 min, 3,500 × *g*, 4°C), and the pellet was resuspended in ice-cold lysis buffer (50 mM Tris, pH 7.5, 500 mM NaCl, 10 mM MgCl_2_, 10% glycerol, 5 mM β-mercaptoethanol [β-ME]) with 1 mM phenylmethylsulfonyl fluoride (PMSF), 10 units/mL DNase and RNase, 0.2% Triton, 5 mg lysozymes, and cOmplete protease inhibitors (1 tablet per 50-mL suspension). The cell suspension was homogenized and lysed using a cell disrupter (Avestin Emulsiflex C3) for 5 min at 8°C. Cell debris was removed by centrifugation (45 min, 14,000 × *g*, 4°C), and the supernatant was loaded onto an immobilized-metal affinity chromatography column (IMAC) (Ni-nitrilotriacetic acid [NTA] agarose; Qiagen). To obtain RNA-free N, we washed the Ni-bound protein with lysis buffer supplemented with 3 M urea and 1 M NaCl prior to imidazole washes. His-tagged N^fl^ was eluted with 300 mM imidazole in 50 mL binding buffer (50 mM Tris, pH 7.5, 500 mM NaCl, 10 mM MgCl_2_, 10% glycerol, 5 mM β-mercaptoethanol). Then, the sample was subjected to TEV protease digestion at a 1:20 protease-to-protein ratio for 16 h at 4°C while being dialyzed against the IMAC binding buffer to reduce the imidazole concentration. The N^ΔN45^ was obtained by trypsin digestion (1:1,000 protease-to-protein ratio) instead of TEV digestion. The digested protein was reloaded onto the IMAC, and the unbound fraction was collected. Washes with 20 mM, 50 mM, and 350 mM imidazole were also collected and analyzed by SDS-PAGE. Digested N proteins (N or N^ΔN45^) were further purified using size exclusion chromatography (SEC) (Superdex 200 10/300 GL; GE Healthcare) preequilibrated with 20 mM Tris, pH 7.5, 150 mM NaCl, and 5% glycerol. Peak fractions were analyzed on 15% SDS-PAGE gels. Relevant fractions were collected and concentrated using a 10-kDa-cutoff spin concentrator (Amicon, Millipore). The samples at concentrations of 6.5 g/L and 7.3 g/L (N^fl^ and N^ΔN45^, respectively) were then aliquoted, flash-frozen in LN_2_, and stored at −80°C.

### Crystallization, data collection, and structure determination.

All crystals were grown by the hanging-drop vapor diffusion method at 16°C. Unfortunately, N^fl^ failed to produce crystals suitable for X-ray diffraction experiments. For determining the structure of RNA-free N, FMV N^ΔN45^ at 7.3 g/L was mixed with a reservoir solution containing 3.45 mM sodium formate, pH 7.0, in a 1:1 protein-to-reservoir solution ratio. Crystals with rectangular-cuboid morphology grew within a week and reached their final size of 400 by 200 by 100 μm 2 weeks after drop setup. These crystals were indexed to the P2_1_ space group. Since the reservoir condition is a cryoprotectant, crystals were mounted on freezing loops and flash frozen in LN_2_. For derivatization, N^ΔN45^ was co-crystallized with 1 mM ethyl-mercury phosphate supplemented with the reservoir solution described above.

N^ΔN45^ and 42-mer ssRNA [poly(U)] were coincubated for 1 h at room temperature for N-ssRNA complex crystallization. The N^ΔN45^-ssRNA complex at 7 g/L was mixed with a reservoir solution of 20% polyethylene glycol 3350 (PEG 3350), 0.2 M magnesium formate, pH 5.9, at a 1:1 protein-to-reservoir solution ratio. Crystals were observed 3 to 7 days after drop setup and grew to a final size of 200 by 250 by 300 μm. Crystals were harvested and flash frozen in LN_2_ in reservoir solution supplemented with 2 M l-proline as a cryoprotectant. These crystals belonged to the P2_1_2_1_2_1_ space group (Table 1). Data from all crystals were collected at 100°K on a Pilatus detector at different synchrotron facilities (Table 1). We used XDS (23, 24) for indexing, integration, and data reduction. We obtained phase information using single isomorphous replacement using anomalous signal (SIRAS) with the PHENIX suite (33). Automatic model building was performed by using the AutoBuild module in PHENIX, followed by iterative cycles of manual building using Coot (34) and refinement. Finally, the structure was refined with PHENIX against a 2.3-Å native data set (Table 1). Subsequently, this model (only the globular core) was used as a search model in PHENIX for a molecular replacement experiment to determine the crystal structure of the FMV N^ΔN45^-ssRNA complex. A density for the 42-nucleotide chain was observed, and the oligonucleotide was manually built using Coot. The N^Δ45^-ssRNA crystals belonged to the P2_1_2_1_2_1_ space group. The N and C arms were built manually, and the structure was refined to 2.5 Å (Table 1). All molecular graphics images were produced using PyMOL (PyMOL Molecular Graphics System, version 1.8; Schrödinger, LLC).

### MALDI-TOF analysis.

Purified FMV N protein samples were dialyzed overnight against double-distilled water to remove salts. We made serial dilutions of purified N^ΔN45^ in a sinapinic acid matrix using 10 mg sinapinic acid, 500 μL deionized water, 500 μL acetonitrile, and 0.3% trifluoroacetic acid (TFA). Samples were analyzed using Bruker’s Autoflex Speed matrix-assisted laser desorption ionization–time of flight (MALDI-TOF) instrument.

### Fluorescence polarization.

A series of 12 2-fold serial dilutions of FMV N protein was made in black opaque Eppendorf tubes. 42-base ssRNA poly(U)–6-carboxyfluorescein (FAM) was added to each sample to a final RNA concentration of 1 μM. Samples were then incubated at 37°C for 20 min (25, 28). Triplicates of the samples were loaded onto Greiner flat-bottom black 384-well plates. The samples were analyzed using the Spark multimode microplate reader (Tecan) using the fluorescence polarization module. Evaluation of the results was performed using GraphPad Prism version 9.0.2 (www.graphpad.com).

### Isothermal titration calorimetry.

Isothermal titration calorimetry (ITC) was performed using the MicroCal PEAQ-ITC instrument (Malvern Panalytical). The ssRNA 42-mer (25 μM) was titrated into purified N^ΔN45^ (10 μM). Both protein and RNA samples were in 20 mM Tris, pH 7.4, 150 mM NaCl, 5% glycerol, and 5 mM β-ME. All measurements were performed at 25°C. The experiment setup included 19 injections of 1.0 μL with an injection duration of 2 s. Data were fitted according to a single-site binding model using the MicroCal PEAQ-ITC Control software, version 1.40 (25, 28).

### Phylogenetic analysis of nucleoproteins from the *Bunyavirales* order.

The nucleoprotein sequences of representative members of the *Bunyavirales* order were obtained from the NCBI archive. The nucleotide sequences were aligned using multiple alignment using Fasta Fourier transform (MAFFT) (35). The neighbor-joining phylogenetic tree was generated with Jalview calculation using the BLOSUM62 matrix (36). The dendrogram was exported and visualized using the interactive Tree of Life tool, version 6 (iTOL) (37, 38). For the structure-based sequence alignment analysis, the PDB entries of N proteins from various bunyaviruses (PDB identification codes [IDs] 4J1J, 4JNG, 4IJS, 5E04, 5E06, 5FSG, 4H5P, 4J4W, 4J4X, 4J4R, 5A97, 4XZC, 4XZ8, 4AQG, 5FVA, 4BGP, and 5Y6J) were aligned to the globular domain of FMV N using the MUSCLE software (39), and a phylogenetic tree was generated. To obtain structure-based clustering of N proteins, we subjected the phylogenetic tree to principal component analysis using GraphPad Prism, version 9.0.2 (www.graphpad.com).

### Data availability.

Atomic coordinates and structure factors for the reported crystal structures have been deposited in the Protein Data Bank under PDB IDs 8AX4 and 8AXF (N^ΔN45^ in its RNA-free form and N^ΔN45^ in complex with the 42-nt ssRNA, respectively).
