# Supplementary material for: RNA Encapsulation Mode and Evolutionary Insights from the Crystal Structure of Emaravirus Nucleoprotein
Source: Microbiol Spectr. 2023 Apr 11;11(3):e05018-22. doi: 10.1128/spectrum.05018-22 (PMC10269810; doi:10.1128/spectrum.05018-22)
Supplement: Supplemental file 1 — Supplemental material. Download spectrum.05018-22-s0001.pdf, PDF file, 7.1 MB [file spectrum.05018-22-s0001.pdf]

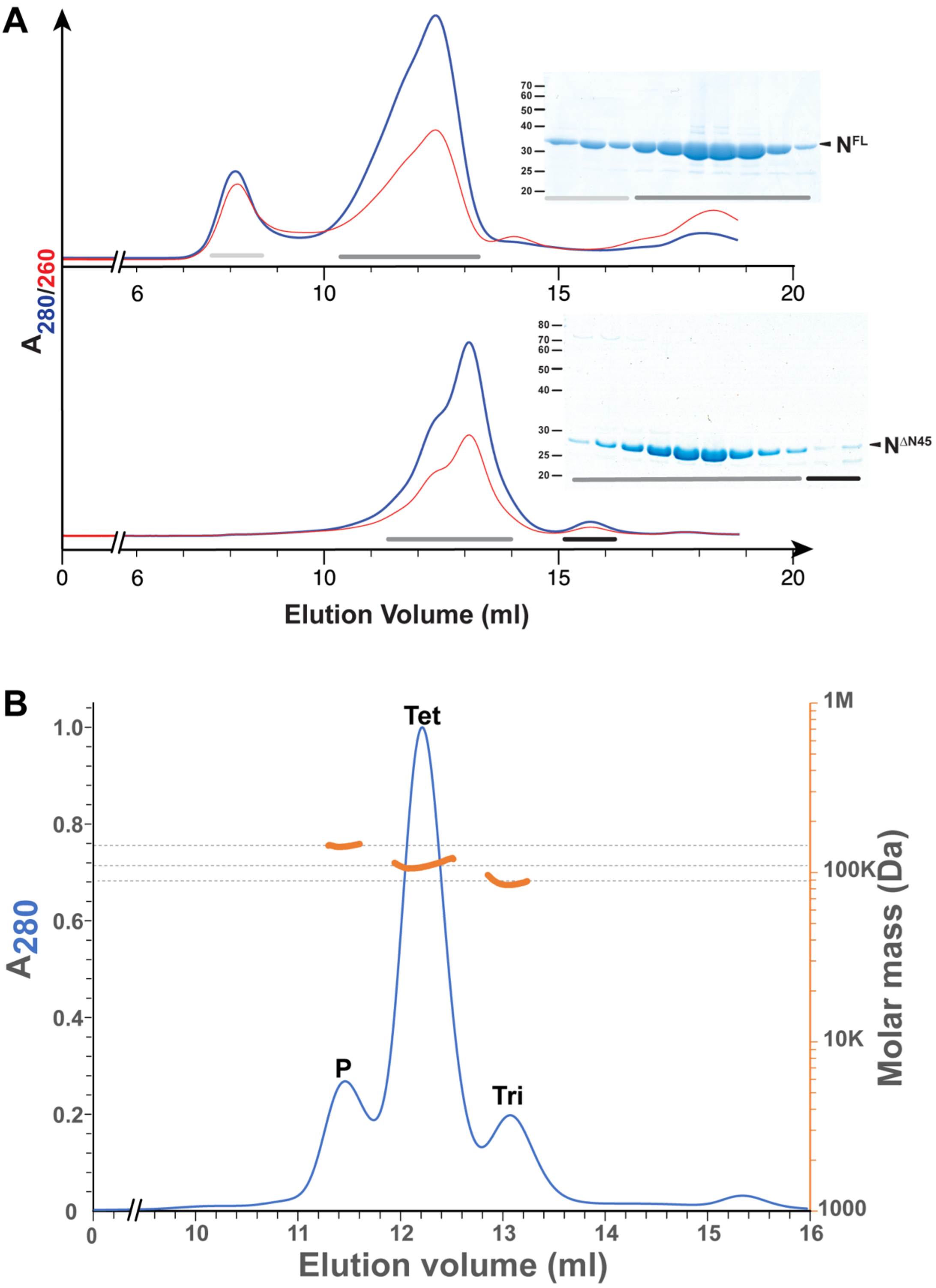

Supplementary Figure S1. Purification of FNV N. (A) The profile of the final Size Exclusion Chromatography (SEC) step of N^{fl} (top) and $N^{\Delta N45}$ (bottom). In the insets ae the corresponding SDS-PAGE analysis. Grey bars indicate the peaks that correspond with the SDS-PAGE samples. (B) SEC-MALS analysis of the purified FMV N. In blue line is the A280 signal and in orange is the MALS calculated molecular mass. Grey dashed lines indicates 90, 120 and 150 KDa.

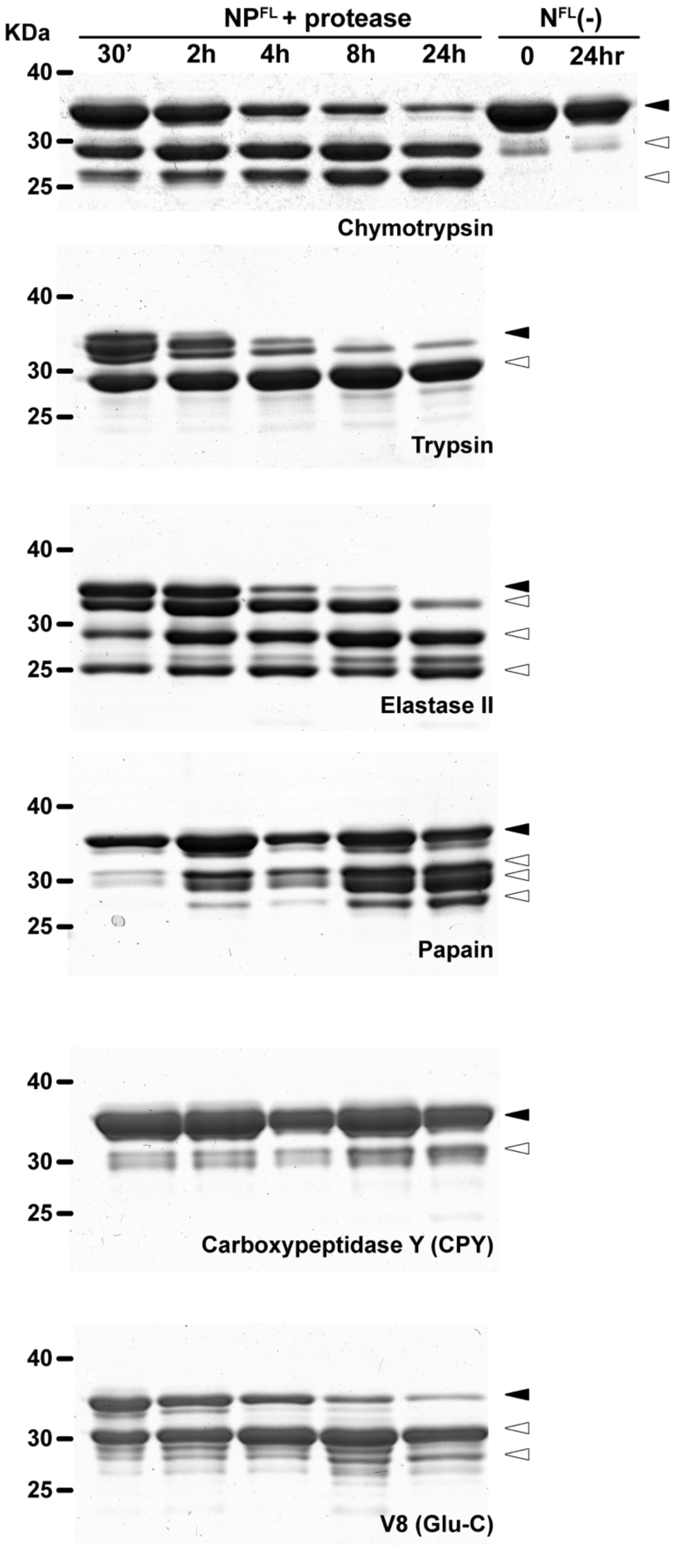

Supplementary Figure S2. Limited proteolysis of FNV N. SDS-PAGE analysis (Coomassie stained) of limited proteolysis experiments of FMV N^{fl} with proteases as indicated per gel. Black arrow heads indicate the relative migration of the uncut N^{fl} and white arow heads indicate the digestion's main products. Reaction duration is indicated as per lane.

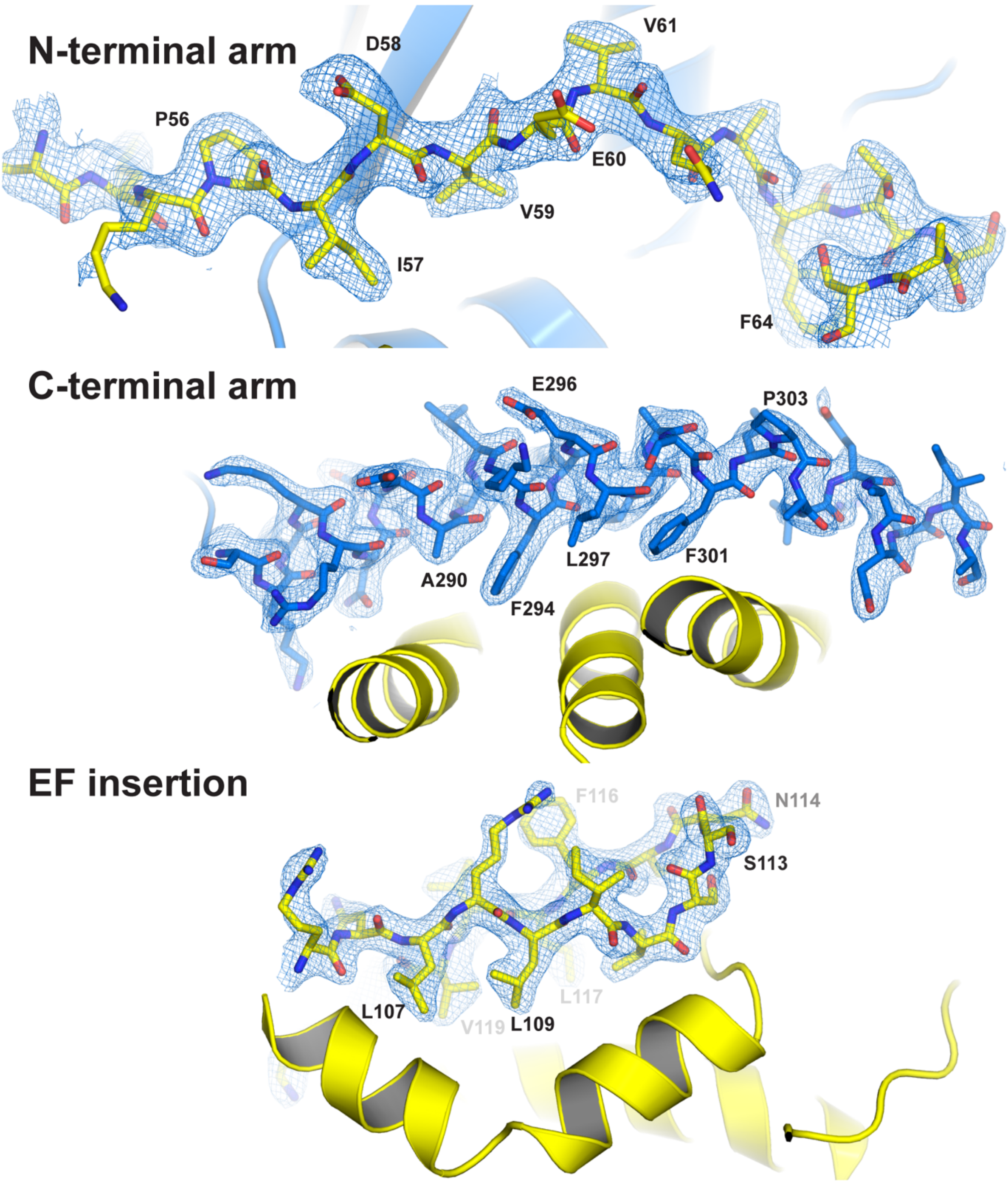

Supplementary Figure S3. Electron density maps for different regions of $N^{\Delta N45}$ structure. A 2.28 Å resolution 2Fo-Fc electron density maps at 1σ are shown as a blue mesh. Atoms covered by the map are shown in a sticks representation whereas the rest of the model is in cartoon representation.

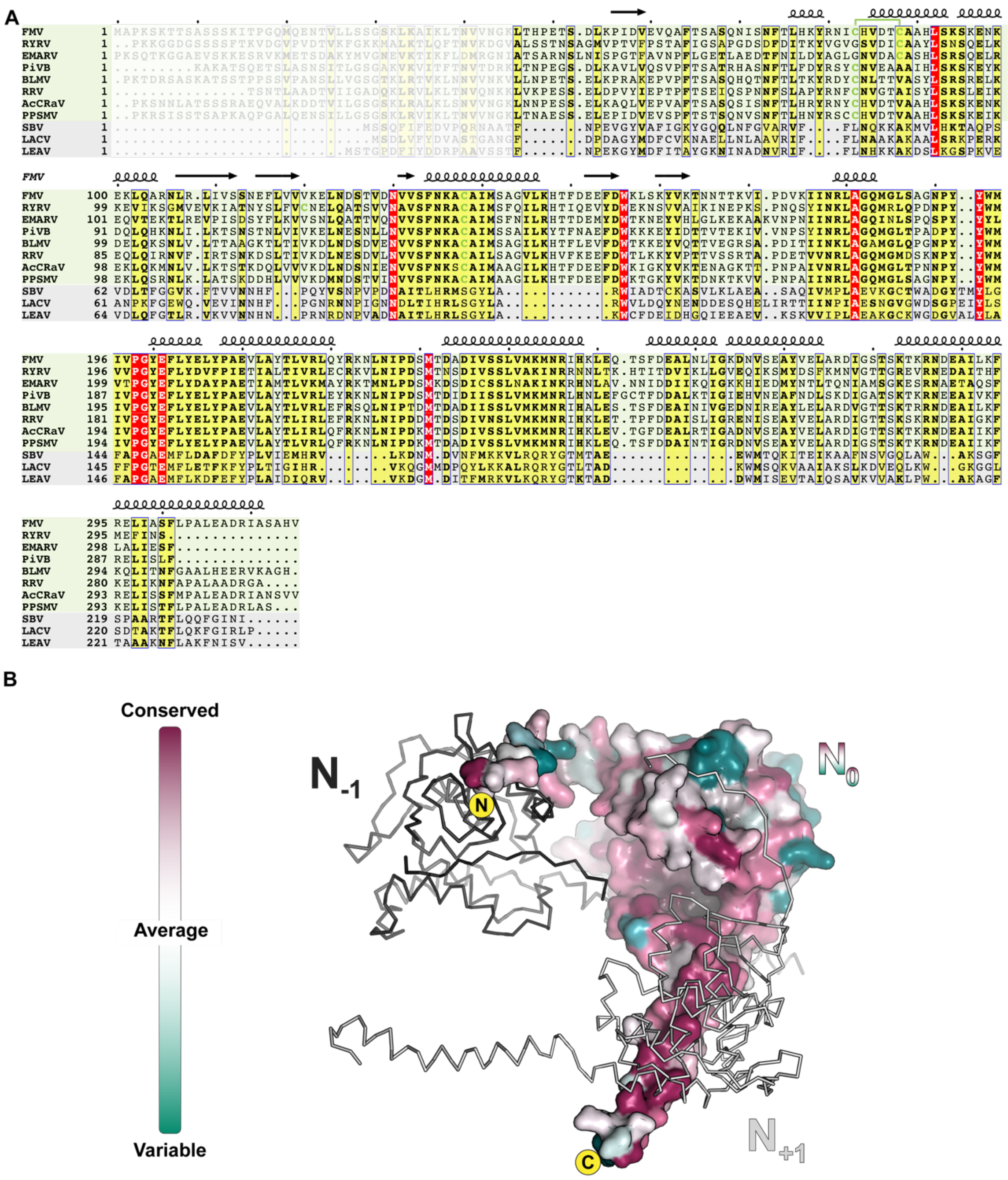

Supp. Figure S4

Supplementary Figure S4. Sequence-based conservation analysis of N proteins from *Fimoviridae* and *Peribunyavirdae* families (A) A Multiple sequence alignment of N proteins from members of the *Fimoviridae* (green shading) and *Peribunyaviridae* (grey shading) families. Secondary structure composition of FMV N is above the the FMV sequence (black coiled stroke – α Helix, black arrow – β strand, green line – disulfide bond). (B) ConSurf analysis (https://consurf.tau.ac.il/consurf_index.php) of FMV N protomer using the sequence alignment in A. Protomers are labelled N_{-1}, N_0, and N_{+1}. Conservation scores colour scheme is represented by the bar on the left.

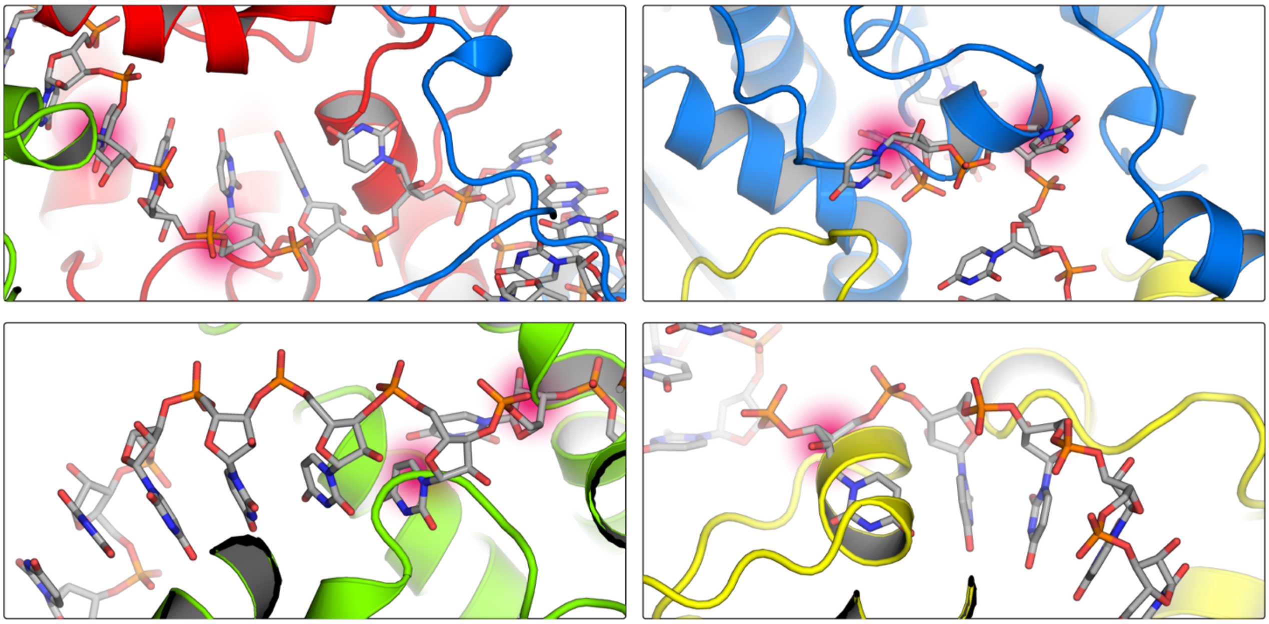

Supplementary Figure S5. The RNA-free N tetramer structure cannot accommodate 42-nt RNA. The RNA -free N structure was superimposed on the RNA-bound N. In the figure, the RNA is shown with the apo-N structure. Clashes between N and the RNA backbone in all four protomers are highlighted with a magenta shading (The protein’s colour scheme is as in figure 2A).

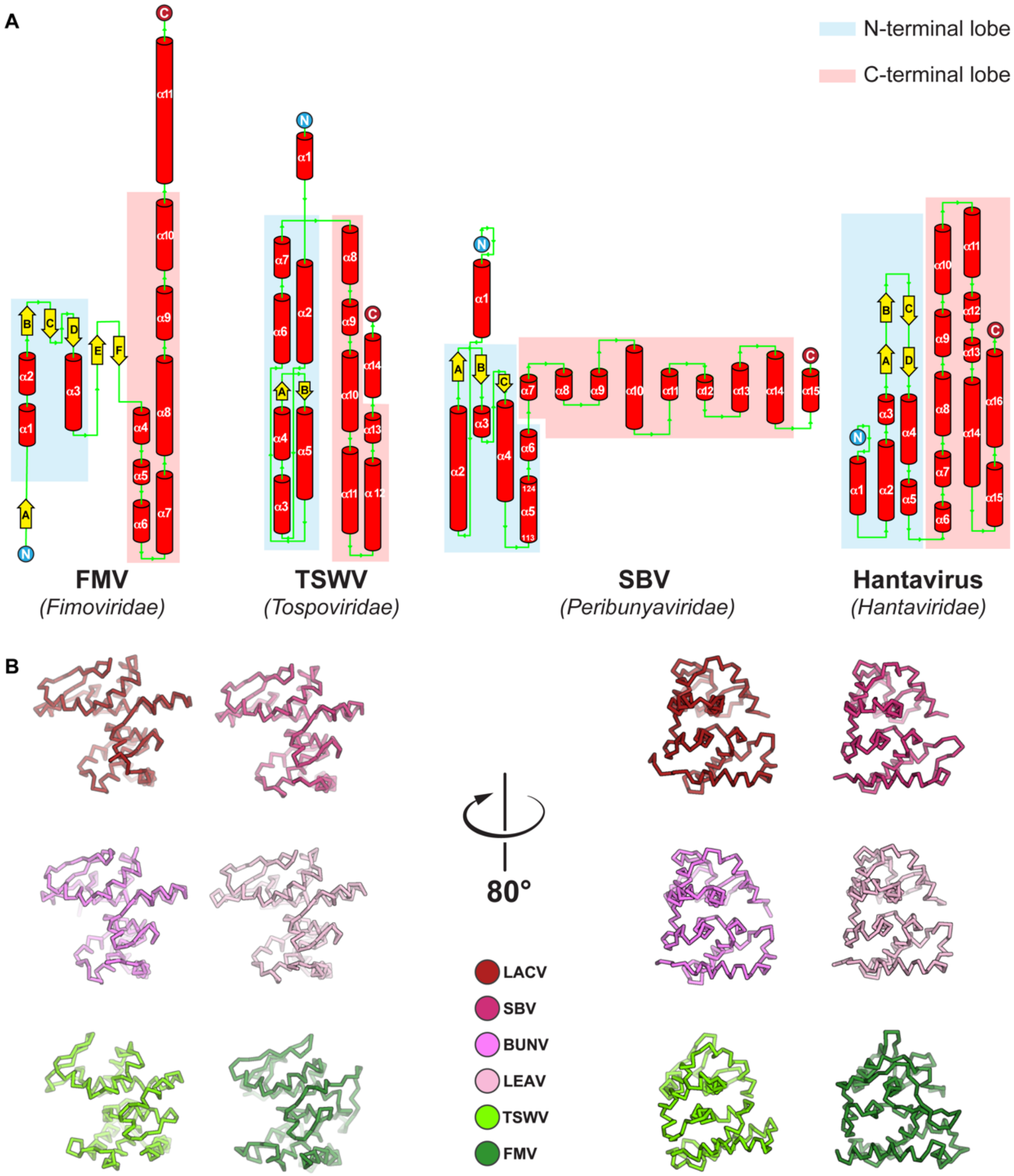

Supplementary Figure S6. Structural comparison of FMV N with N proteins from other bunyaviruses. (A) Topology diagram of different bunyaviruses. Red cylinders represents α Helices, yellow arrows - β strands, blue and red shading for N- and C- terminal lobes of the proteins core ,respectively. (B) Ribbon representation of superimposed N proteins from plant infecting bunyaviruses (shades of green) with N proteins from the *Peribunyaviridae* members (shades of crimson/pink). LACV- La-crosse virus, SBV-Schmalenberg virus, BUNV-Bunyamwera virus, LEAV-Leanyer virus, TSWV-tomato spotted wilt virus, FMV-fig mosaic virus.

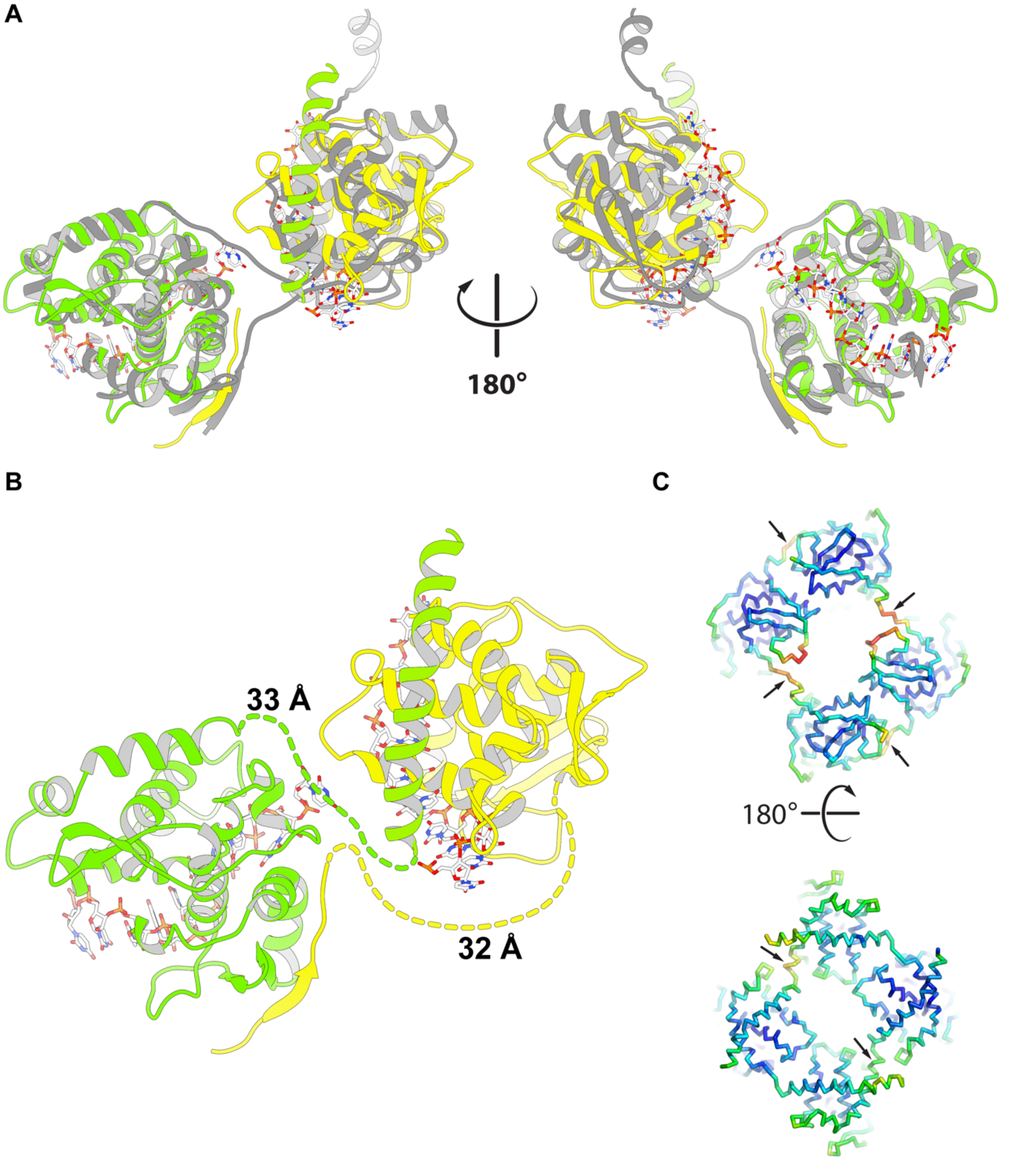

Supplementary Figure S7. Superposition of FMV N structure onto the pseudo atomic model of BUNV RNP. (A) The composite FMV N building block models (green and yellow) are super imposed onto the BUNV RNP model (PDB ID 7AOY, grey). The RNA bound to FMV N is in sticks representation. (B) An illustration of the connectivity between building blocks of the FMV RNP. The dashed lines represent the relevant gaps and their distances. (C) FMV N tertramer in its RNA bound conformation in a ribbon representation and coloured according to the Cα temperature factor (b-factor). Cold (blues and greens) colours represents low b-factor (rigid) whereas warm colours (yellows and reds)represent high b-factor regions (flexible).
